# Immunohistochemical Analysis of Inter-Alpha-Trypsin Inhibitor Heavy Chain 2 and Enolase 1 in Canine Mammary Tumors: Associations with Tumor Aggressiveness and Prognostic Significance

**DOI:** 10.3390/vetsci12020110

**Published:** 2025-02-02

**Authors:** Luadna dos Santos e Silva, Pedro Henrique Fogaça Jordão, Beatriz Castilho Balieiro, Laura de Souza Baracioli, Daniela Farias de Nóbrega, Adriana Alonso Novais, Luiz Gustavo de Almeida Chuffa, Debora Aparecida Pires de Campos Zuccari

**Affiliations:** 1 Molecular Investigation of Cancer Laboratory (MICL), Department of Molecular Biology, Faculty of Medicine of São José do Rio Preto (FAMERP), São José do Rio Preto 15090-000, Brazil; lua_dna@outlook.com (L.d.S.e.S.); bia-balieiro@hotmail.com (B.C.B.); laura.baracioli@hotmail.com (L.d.S.B.); 2 Biochemistry and Molecular Biology Research Center (NPBIM), Faculty of Medicine of São José do Rio Preto (FAMERP), São José do Rio Preto 15090-000, Brazil; pedro.hf.jordao@hotmail.com; 3 Pat Animal Laboratory, São José do Rio Preto 15070-000, Brazil; danielanobrega@patanimal.com.br; 4 Institute of Health Science (ICS), Universidade Federal de Mato Grosso (UFMT), Sinop 78550-728, Brazil; adriana.novais@ufmt.br; 5 Department of Structural and Functional Biology, Institute of Biosciences, UNESP, São Paulo State University, Botucatu 18618-689, Brazil; luiz-gustavo.chuffa@unesp.br

**Keywords:** bitches, mammary tumors, extracellular vesicles (EVs), biomarkers, prognosis

## Abstract

Mammary neoplasms in dogs are a common clinical concern, especially in middle-aged and older intact females. These tumors share similarities with human breast cancer in terms of histology, disease progression, and risk factors, making dogs a relevant model for breast cancer research. The search for biomarkers in canine mammary tumors is essential to understand tumor progression and identify potential therapeutic targets. This study investigated the expression of two potential biomarkers—Inter-Alpha-Trypsin Inhibitor Heavy Chain 2 (ITIH2) and Enolase 1 (ENO1)—in the mammary glands of healthy and tumor-bearing dogs using immunohistochemistry. Both proteins were identified in previous proteomic analyses of extracellular vesicles derived from the plasma of healthy and tumor-bearing dogs. A total of fifty-one canine mammary tissue samples were analyzed and categorized into three groups: (i) the control group, composed of five samples of normal mammary tissue without neoplasia; (ii) benign tumors, composed of nineteen samples of benign mixed tumors; and (iii) malignant tumors, which included six carcinomas in grade 1 mixed tumors, five carcinomas in grade 2 mixed tumors, thirteen solid carcinomas of grade 3, one papillary carcinoma, and two tubular carcinomas. Regarding the intensity of staining, quantified by histoscore, there were no significant differences in the comparison between the groups; for ITIH2, the *p*-value was 0.33, and for ENO1, the *p*-value was 0.57. Regarding the predictive potential of their respective ROC curves, the proteins demonstrated low predictive power in canine mammary tumors. These findings indicate that neither ITIH2 nor ENO1 demonstrated strong prognostic value in this setting, as demonstrated by their moderate AUC values, wide confidence intervals, and lack of statistical significance. However, this study found distinct tissue localization patterns for ITIH2 and subcellular localization for ENO1. As an additional way to examine possible associations of these proteins with epithelial–mesenchymal transition, the ZEB1 antibody was tested by both single and double immunohistochemistry, demonstrating a tendency to be more intensely expressed in the malignant group and tending to be associated with ENO1 in canine mammary tumors.

## Simple Summary

Mammary tumors in dogs are commonly seen in middle-aged and older females, particularly in those that are not spayed. These tumors share many similarities with human breast cancer, making dogs a useful model for studying the disease. This study focuses on two potential biomarkers—Inter-Alpha-Trypsin Inhibitor Heavy Chain 2 (ITIH2) and Enolase 1 (ENO1)—which have been identified in previous research as relevant to canine mammary tumors. We used immunohistochemistry to analyze tissue samples from healthy dogs and those with benign or malignant tumors. The results showed specific patterns of expression for both ITIH2 and ENO1, suggesting that these proteins may play important roles in tumor development. This study highlights their potential as biomarkers for diagnosing and treating mammary tumors, both in dogs and, possibly, in human breast cancer.

## 1. Introduction

Mammary neoplasms are the most common group of tumors encountered in veterinary practice, exhibiting a biological behavior that ranges from benign to highly malignant. Metastases are reported in 25 to 50% of cases, emphasizing the aggressive potential of these tumors. Mammary cancer in dogs serves as an exceptional model for studying breast cancer in women due to striking similarities in tumor histology, disease progression, behavior, associated risk factors, and hormone receptor profiles [1].

These neoplasms often originate as benign lesions. However, under certain conditions, they may accumulate genetic and epigenetic changes that drive their progression to malignancy. On the other hand, malignant mammary tumors typically exhibit an aggressive phenotype from the moment of diagnosis, including invasiveness and metastatic potential. Studies report that approximately 25% of dogs experience the development of new nodules following surgical removal of initial tumors. Interestingly, these subsequent nodules may display malignant characteristics, even when the primary lesion was histologically classified as benign [1]. As previously reported [2], hormonal stimulation—often associated with recurrent pseudocyesis—can exacerbate this process. In cases where tumors are not surgically removed promptly, hormonal influence may lead to tumor disorganization, the accumulation of alterations and mutations, and ultimately, a worse prognosis. Delays in surgical excision increase the likelihood that these tumors will undergo malignant transformation, further complicating treatment and prognosis [3].

Mammary neoplasms mainly affect middle-aged and elderly female dogs that have not been spayed. Breeds such as Poodles, Dachshunds, Yorkshire Terriers, Cocker Spaniels, German Shepherds, Boxers, Fox Terriers, and mixed-breed dogs are predisposed to developing these tumors. Malignant mammary neoplasms, particularly carcinomas, are characterized by an aggressive inflammatory process, rapid growth, regional lymphatic infiltration, and metastasis to distant tissues [3]. The lymphatic system is the primary route of dissemination, with the lungs being the most commonly affected site in metastatic cases. Consequently, thorough evaluation using complementary diagnostic exams is essential for accurate staging and treatment planning [4].

Cytological criteria, such as variation in nuclear size, prominent nuclei, altered nuclear-to-cytoplasmic ratios, and variations in the number, shape, and size of the nucleoli are important predictive factors of malignancy. However, one of the main challenges in obtaining an accurate cytological diagnosis in mammary neoplasms in dogs is the significant heterogeneity of tumor tissue. This heterogeneity arises from the presence of necrotic and inflamed regions, which can distort cellular morphology, as well as from distinct niches of tumor cells displaying varying levels of genetic and phenotypic alterations. Such variability complicates the interpretation of cytological features and may obscure the distinction between benign and malignant characteristics [5].

Dogs have five pairs of mammary glands, and the incidence of multiple mammary tumors has been reported to reach as high as 60%. Despite the relatively high occurrence of synchronous mammary tumors, the relationship between these tumors remains poorly understood. Most reports suggest that they are independent primary tumors with distinct histopathological features, leading to the recommendation that all tumors be biopsied to assess malignancy. Therefore, benign and malignant tumors are generally considered separate entities, with the former not acting as precursors to the latter. However, recent studies challenge this assumption. One publication reported an increased risk of developing new malignant primary tumors in other mammary glands in dogs with a previous diagnosis of mammary carcinoma compared to those with benign tumors. Additionally, another study reported a high incidence of concurrent carcinoma in situ in dogs diagnosed with carcinomas [6].

The search for biomarkers in canine mammary tumors is particularly relevant for predicting tumor progression in both species, providing insights into shared mechanisms of carcinogenesis between dogs and humans. These similarities emphasize the importance of identifying early diagnostic biomarkers for metastasis and prognostic markers that can guide clinical practice, ultimately improving patient outcomes in both species.

## 2. Inter-Alpha-Trypsin Inhibitor Heavy Chain 2 (ITIH2)

ITIH2 belongs to a family of structurally related plasma serine protease inhibitors. These proteins contribute to matrix stability by covalent binding to hyaluronan and play significant roles in inflammation and cancer metastasis prevention [7,8].

ZEB1 is a hyaluronan-binding protein, and ITIH2 is involved in constructing a hyaluronan network. It is secreted by mesenchymal lung cancer cells when co-cultured with cancer-associated fibroblasts. The protein transcriptionally regulates 1, an epithelial–mesenchymal transition (EMT)-inducing transcription factor. Together, the ZEB1-regulated EMT signaling contributes to establishing a pro-metastatic hyaluronan network in the tumor microenvironment, presenting a novel strategy to target this network and suppress lung cancer progression and metastasis [9].

Overall, underexpression of ITIH2 is associated with longer overall survival of colorectal cancer patients; the overexpression of ITIH2 was associated with poor overall survival. ITIH2 is enriched in normal liver tissues and liver cancer tissues. However, the ITIH2 gene was not detected in tumor cells in colon cancer [10].

### 2.1. ITIH2 and Breast Cancer in Women

Normal breast epithelial cells clearly express ITIH2, whereas its expression is consistently lost or strongly decreased in invasive breast cancer. Patients with abundant ITIH5 expression have better clinical outcomes compared with those with reduced expression in node-negative invasive breast cancer. The strong correlation between ITIH2 and ITIH5, especially since they are located on the same chromosome, suggests that these molecules interact in their metastasis-suppressive and metastasis-repressive properties [11].

### 2.2. ITIH2 and Mammary Tumors in Bitches

The role of ITIH2 in canine mammary neoplasms remains unexplored, representing a promising opportunity to expand knowledge on comparative biomarkers and tumor mechanisms.

## 3. Enolase 1 (ENO1)

Enolase 1 is a key enzyme in glycolysis, responsible for converting 2-phosphoglycerate into phosphoenolpyruvate, a fundamental step in glucose breakdown for energy production. This enzyme is widely expressed in various tissues, primarily located in the cytoplasm of cells [12]. Beyond its metabolic role, ENO1 functions as a plasminogen receptor on the surface of cancer cells, facilitating extracellular matrix degradation, cell invasion, and metastasis. In the cytoplasm, it catalyzes the glycolytic conversion between 2-phosphoglycerate and phosphoenolpyruvate. On the cell surface, it mediates plasminogen activation and extracellular matrix degradation, promoting cell migration and metastasis. Additionally, in the nucleus, ENO1 acts as a C-Myc promoter-binding protein (MBP-1) [13,14].

### 3.1. ENO1 and Breast Cancer in Women

ENO1 has been implicated in several cancer types, showing relevance in oncological studies. Its overexpression is associated with overall survival in patients, including those with breast cancer.

In an experimental study [15], the effect of reduced Enolase 1 expression in human umbilical vein endothelial cells (HUVECs)/MDA-MB-231 cells on the response to hypoxia and the possible mechanisms involved were investigated. After Enolase 1-transfected breast cancer cells were injected into nude mice, tumor growth significantly decreased, and tumor volume and weight both reduced. After radiotherapy treatment, tumor size decreased significantly in both groups, and the greatest reduction was observed in the transfected group. Reduction in Enolase 1 expression significantly decreases the response to hypoxia and increases the sensitivity of cells to radiotherapy. Other studies have also demonstrated that tumor-conditioned medium from the breast carcinoma cell line, MDA-MB-231 cells, led to upregulated expression of Enolase 1 in HUVECs, elevated reproductive and antiapoptotic capacity of endothelial cells, increased cell cycle progression, and improved angiogenesis in vitro [16,17].

Overexpression of ENO 1 has also been associated with tamoxifen resistance in breast cancer, in addition to reduced overall survival in cancer patients. The proteins E6AP (ubiquitin ligase E6-associated protein) and ENO1 interact predominantly in the cytoplasmic periphery of breast cancer cells. E6AP has been shown to target ENO1 for degradation, highlighting the importance of this interaction in oncological contexts, especially in breast cancer [18].

The ENO1 supports anaerobic proliferation via the Warburg effect and promotes cancer invasion through its surface expression as a plasminogen receptor. Its dual roles in metabolism and metastasis make it a promising target for cancer therapy [19].

### 3.2. ENO1 and Mammary Tumors in Bitches

There is only a single published study on ENO1 and canine mammary tumors, and that study showed that ENO1 overexpression was detected only in canine mammary carcinoma tumor cells and significantly correlated with shorter 5-year cause-specific survival. The results of age-adjusted Cox regression analysis also indicated that ENO1 overexpression was significantly and independently associated with shorter cause-specific survival. In contrast to the results of the human breast cancer study, the findings suggested that ER positivity was not associated with ENO1 overexpression in canine mammary carcinoma [20]. Although there has been a single published study on Enolase 1 in canine mammary tumors that used the immunohistochemical technique, it did not focus on the various prognostic regulations that this protein can influence depending on the labeling site, as ours did.

The proteins ITIH2 and Enolase1 (ENO1) were found to be overexpressed in extracellular vesicles (EVs) extracted from the plasma of bitches with malignant mammary tumors in a previous proteomic study carried out by our research group. Therefore, the aim of our study is to validate these proteins in the mammary gland tissue of healthy bitches and those with benign and malignant mammary tumors to investigate whether they have prognostic potential for these tumors.

## 4. Materials and Methods

### 4.1. Samples and Histological Processing

The tissue blocks selected for this study were previously submitted to histopathological evaluation (hematoxylin–eosin stain) by the clinic from which they were acquired, focusing on cellular morphology, lymphatic invasion, and metastasis to identify what type of tumor each sample was, whether benign or malignant; this was carried out by adhering to the criteria established by the Consensus on Mammary Tumors in Dogs and Cats [21]. A total of fifty-one canine mammary tissue samples were analyzed and categorized into three groups: (i) the control group, consisting of five samples of normal mammary tissue without neoplasia; (ii) benign tumors, consisting of nineteen samples of benign mixed tumors; and (iii) malignant tumors, which included six grade 1 carcinomas in mixed tumors, five grade 2 carcinomas in mixed tumors, thirteen grade 3 solid carcinomas, one papillary carcinoma, and two tubular carcinomas. All samples were obtained from a veterinary pathology laboratory tumor biobank, ensuring standardized preservation and accessibility for this study. Regarding the test with ZEB1 performed, only 10 tumor samples were used (4 benign and 6 malignant, with the malignant ones subdivided into good and reserved prognosis).

### 4.2. Positive and Negative Controls

**Figure 1:**
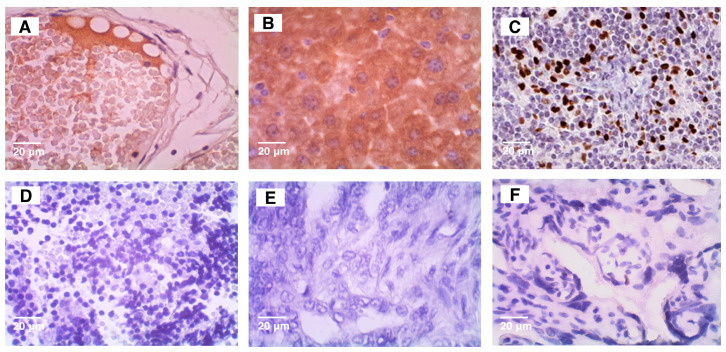
Photomicrographs of the positive and negative controls of ITIH2, ENO1, and ZEB1. (**A**)—Positive control of ITIH2 (fallopian tube) and (**D**)—negative control (tonsil); (**B**)—Positive control of ENO1 (liver) and (**E**)—negative control (antibody suppression in dog mammary gland tissue); (**C**)—Positive control of ZEB1 (lymph node) and (**F**)—negative control (placenta).

Positive and negative controls for each antibody used (Figure 1) were obtained according to The Atlas Human Protein website.

### 4.3. Single Immunohistochemical Labeling

For the single immunohistochemical labeling, 4 μm thick tissue sections were prepared using a microtome from the manufacturer Leica (Model 2255, Wetzlar, Germany). The slides were deparaffinized in xylene and rehydrated through a graded alcohol series, followed by incubation in an oven at 60 °C for 3 h. Antigen retrieval was performed using citrate buffer (pH 6.0) for ITIH2 or EDTA buffer (pH 7.4) for ENO1, both heated to 100 °C in a steam cooker (Histo Bath, from the manufacturer Easy Path, São Paulo-SP, Brazil) for 30 min. Endogenous peroxidase activity was blocked using a DAKO peroxide solution for 15 min, and non-specific protein binding was minimized with a 3% BSA solution in PBS for 10 min.

Primary antibodies (anti-ITIH2 at 1:300, anti-ENO1 at 1:3000, and anti-ZEB1 at 1:500) were applied and incubated overnight at 4 °C to ensure specific binding. Afterward, horseradish peroxidase (HRP)-conjugated secondary antibodies were applied for 25 min. Immunoreactivity was visualized using diaminobenzidine tetrahydrochloride (DAB) as the chromogen (1 drop per 1000 μL of DAKO substrate) for 2 min. Finally, the slides were counterstained with hematoxylin for 2 min to enhance cellular structure visualization and mounted for analysis.

### 4.4. Double Immunohistochemical Labeling

The double labeling procedure followed the same initial steps as single labeling, including tissue section preparation, deparaffinization, antigen retrieval, and blocking. The distinguishing feature was the sequential application of two primary antibodies. Following the first immunolabeling, which utilized DAB as the chromogen, an acid block was applied to inactivate residual HRP activity and prevent cross-reactivity with the second antibody.

Next, the second primary antibody was introduced, followed by incubation with a distinct HRP-conjugated secondary antibody and visualization using a contrasting chromogen to differentiate it from the first labeling. Stringent washing steps were performed between each stage to remove residual reagents or minimize nonspecific binding.

This method ensures precise localization and differentiation of multiple target antigens within the same tissue section, facilitating a deeper understanding of co-expressed biomarkers.

### 4.5. Microscopy and Quantification of Immunohistochemical Labeling Intensity

The photomicrographs were captured at 40× magnification on a NIKON ECLIPSE LEICA EC3 microscope, model E200, and using the LAZ EZ Imaging software for Windows, software, version 3.4.0. These images were then analyzed by the IMAGE J [22], using the IHC Profiler plugin, version 1.46r [23] that quantified the DAB and hematoxylin staining, generating three numerical results for each sample. First, the software determined the staining intensity by dividing the pixels into three categories: high positive, low positive, and negative. The values obtained were recorded in an Excel spreadsheet, where the following formula was applied to calculate the staining histoscore (HS) 16, 17, which ranges from 0 to 300: HS = (1 × % low positive cells) + (2 × % positive cells) + (3 × % high positive cells).

### 4.6. Statistical Analyses

Statistical analyses were performed using R [24,25] and GraphPad Prism (v. 5.0). The normality of residuals of quantitative variables was analyzed by the Shapiro–Wilk test, and Levene’s test assessed variance homogeneity. Quantitative data were described as mean ± standard error or median with minimum and maximum values, depending on data distribution. Group comparisons were performed using ANOVA followed by Bonferroni post hoc tests, *t*-tests with Welch’s correction, or Kruskal–Wallis with Dunn’s post hoc, as appropriate. For categorical variables, Fisher’s exact test was applied. Residuals from the chi-square test were assessed to identify significance in the observed distributions. Count data were modeled using a quasi-Poisson regression. All graphs were generated using the ggplot2 package [26] and GraphPad Prism (v. 10.3.1).

### 4.7. Prognostic Classification

The terms “good prognosis” and “reserved prognosis” refer to tumors that, in most cases, tend not to progress to death and those that progress rapidly to death, respectively. For this classification, criteria such as lymphatic invasion, tumor size, morphological aspects of the cells and metastasis were considered.

## 5. Results

### 5.1. Age and Breed

**Figure 2:**
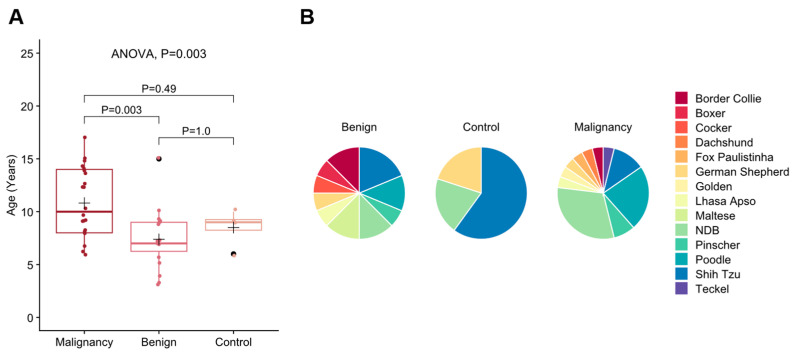
(**A**) Box plot illustrating age distribution across the malignant, benign, and control groups. *p*-values were calculated using ANOVA with Bonferroni post hoc tests. The black cross represents the mean age within each group. (**B**) Proportion representation of breeds within the malignant, benign, and control groups.

This study revealed significant age differences among the groups. The mean age was significantly higher in the malignant group (M = 10.82 ± 0.70) compared to the benign group (M = 7.40 ± 0.68; *p* = 0.003), while no difference was observed between the malignant and control groups (M = 8.5 ± 0.86; *p* = 0.49) or the benign and control groups (*p* = 1.0) (Figure 2A). Regarding breeds, the malignant group included a wide variety of breeds such as Border Collie (n = 01), Dachshund (n = 01), Fox Paulistinha (n = 01), Golden Retriever (n = 01), Lhasa Apso (n = 01), German Shepherd (n = 01), Pinscher (n = 02), Poodle (n = 06), Shih Tzu (n = 03), Teckel (n = 01), and undefined breed (NDB) (n = 08). Similarly, the benign group included Border Collie (n = 02), Boxer (n = 01), Cocker Spaniel (n = 01), Lhasa Apso (n = 01), Maltese (n = 02), German Shepherd (n = 01), Pinscher (n = 01), Poodle (n = 02), Shih Tzu (n = 03), and NDB (n = 02). The control group, however, was composed only of German Shepherd (n = 01), Shih Tzu (n = 03), and NDB (n = 01) (Figure 2B).

### 5.2. Anatomopathological Profile

Frequent necrosis was significantly more common in malignant tumors ($r_{ij}$ = 1.83), while it was less prevalent in the benign group ($r_{ij}$ = −1.73; *p* = 0.027). On the other hand, mild anisokaryosis was observed more frequently in benign tumors ($r_{ij}$ = 2.3), whereas moderate anisokaryosis was predominant in malignant tumors ($r_{ij}$ = 1.71; *p* = 0.005). Furthermore, marked nuclear pleomorphism was significantly associated with malignant tumors ($r_{ij}$ = 1.71) and was notably less frequent in the benign group ($r_{ij}$ = −1.61; *p* < 0.001). However, mild pleomorphism was more common in benign tumors ($r_{ij}$ = 1.66) and less prevalent in the malignant group ($r_{ij}$ = −0.89; *p* = 0.001). Tumor diameter did not differ significantly between the malignant and benign groups (*p* = 0.063), and the presence of vascular invasion and metastases was similar between these groups (Table 1). All sample data are available in the Supplementary Materials.

To analyze the mitotic rate, a regression model with a quasi-Poisson distribution was applied. The mean mitotic rate in the malignant group was approximately 25.33 mitoses per sample (β = 3.23 ± 0.21; *p* < 0.001). In contrast, the benign group exhibited a mean mitotic rate of 0.92 mitoses per sample (β = −3.31 ± 0.75; *p* < 0.001). This indicates a substantial difference, with the mitotic rate between the two groups being approximately 0.04% of that observed in the malignant group. These findings underscore the significantly higher proliferative activity in malignant tumors compared to benign ones.

### 5.3. Protein Expression

**Figure 3:**
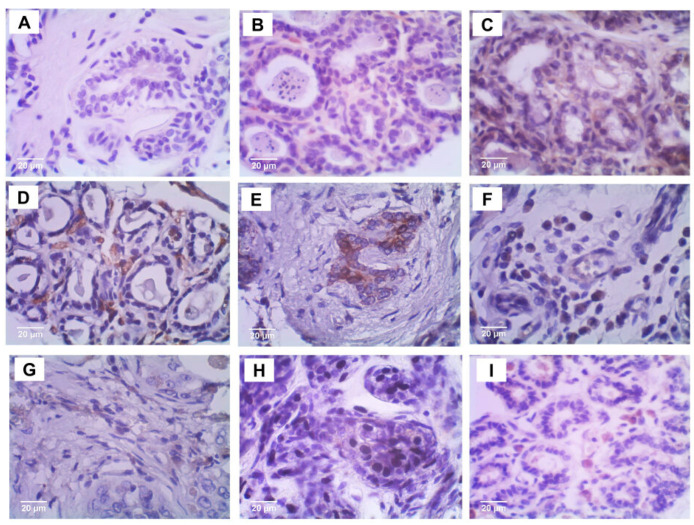
Immunohistochemical staining of ITIH2. Control group (**A**–**C**); benign tumors (**D**–**F**); and malignant tumors (**G**–**I**). Photomicrographs at 20 µm showing weak or absent immunostaining in the cytoplasm.

**Figure 4:**
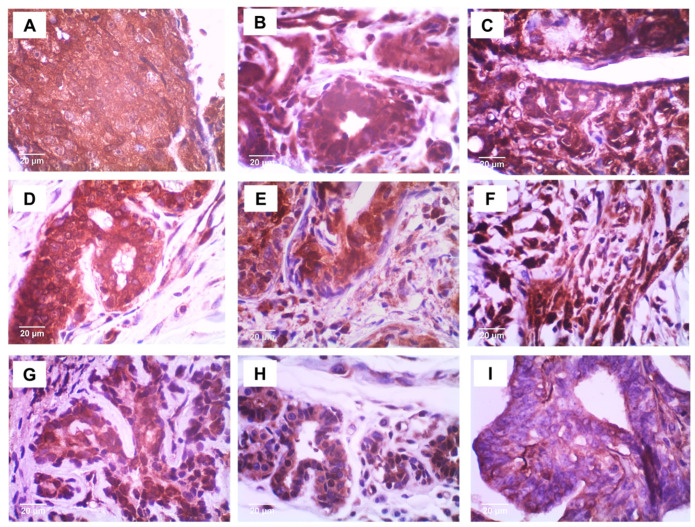
Immunohistochemical staining of ENO1. Control group (**A**–**C**); benign tumors (**D**–**F**); and malignant tumors (**G**–**I**). Photomicrographs at 20 µm showing strong immunostaining in nucleus, cytoplasm, and membrane (**I**).

**Figure 5:**
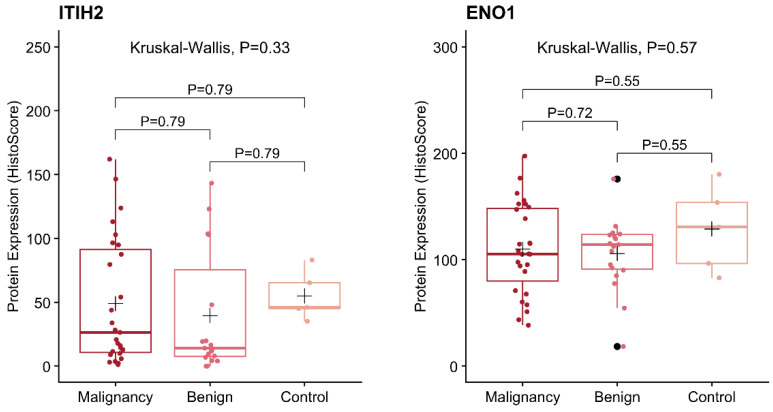
Protein expression (HistoScore) of ITIH2 (Inter-Alpha-Trypsin Inhibitor Heavy Chain 2) and ENO1 (Enolase 1). *p*-values for the Kruskal–Wallis test and Dunn’s post hoc test. The black cross represents the mean.

**Figure 6:**
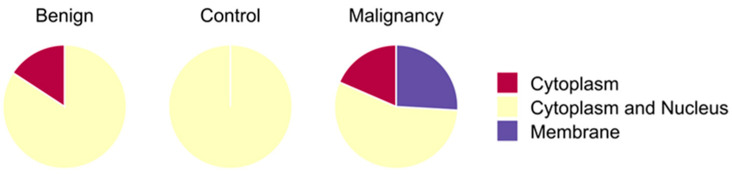
Immunohistochemical localization of ENO1 across cellular compartments.

The protein expression of ITIH2 (Figure 3) and ENO1 (Figure 4) was evaluated, and histoscore quantifications were performed. The expression levels were compared between the malignant, benign, and control groups (Figure 5). However, the immunohistochemical staining patterns did not differ between the groups. Regarding the ENO1 staining sites, it was observed that it was present in several cellular compartments (Figure 6). In the control group, 100% of the stainings were of the cytoplasm and nucleus simultaneously; in the benign group, 84.21% of the stainings were of the cytoplasm and nucleus simultaneously, and 15.79% were of the cytoplasm only; as for the malignant group, there was 18.52% of cytoplasm staining, 55% of simultaneous cytoplasm and nucleus staining, and 25.92% of plasma membrane staining.

**Figure 7:**
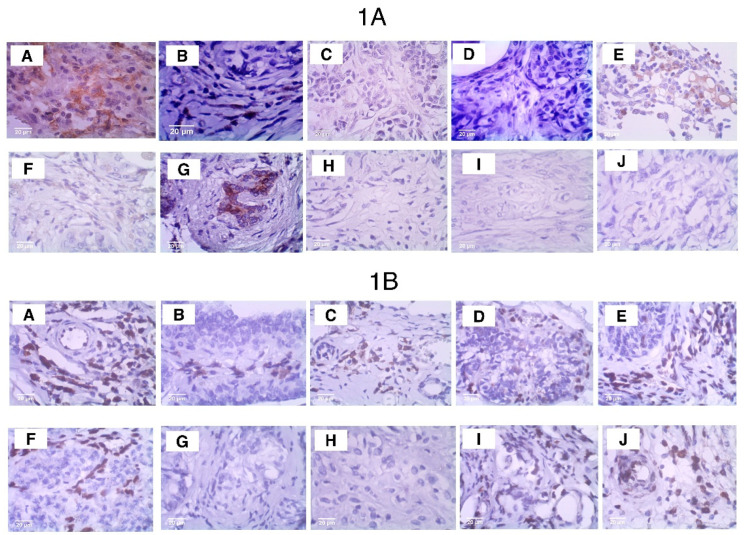
Immunohistochemical comparison of ITIH2 (**1A**) and ZEB1 (**1B**) staining. The tissues in (**1A**) and (**1B**) are identical, differing only by the marker used. Tumor types and grades include carcinoma in mixed tumor grade 2 (**A**); carcinoma in mixed tumor grade 1 (**H**–**J**); tubular carcinoma (**C**); solid carcinoma grade 3 (**E**,**F**); and benign mixed tumor (**B**,**D**,**G**). Metastatic tumors are shown in (**A**,**C**,**E**).

**Figure 8:**
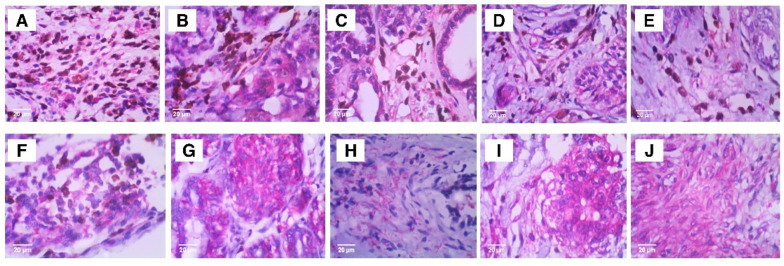
Double immunohistochemical staining showing ENO1 (magenta) and ZEB1 (brown) expression. Tumor types and grades include carcinoma in mixed tumor grade 2 (**A**); carcinoma in mixed tumor grade 1 (**H**–**J**); tubular carcinoma (**C**); solid carcinoma grade 3 (**E**,**F**); and benign mixed tumor (**B**,**D**,**G**).

Immunohistochemistry for the mesenchymal–epithelial transformation marker ZEB1 was performed to analyze its presence or absence in the samples. In cases where ZEB1 was present, the staining intensity was assessed observationally (Figure 7(1A,1B)). Double immunohistochemistry staining for ENO1 and ZEB1 (Figure 8) was performed to evaluate potential similarities or differences in their expression patterns. This analysis aimed to identify shared molecular pathways, mechanisms, or relevant distinctions between tumors with different prognoses.

### 5.4. Test to Evaluate the Association of ITIH2 and ENO1 with ZEB1 in Female Dog Mammary Tumors

Immunohistochemical analysis using the ZEB1 antibody was performed (Figure 7(1A,1B)) on samples (n = 6) of benign and malignant tumors with good prognosis (benign mixed tumor and carcinoma in mixed tumor, grade 1) and on samples (n = 4) of malignant tumors with reserved prognosis (carcinoma in mixed tumor, grade 2; tubular carcinoma; and solid carcinoma, grade 3). This particular analysis involved two observers who individually analyzed each of these 10 samples so that we could then proceed with considerations on staining intensities. When comparing the staining intensities of ITIH2 with those of ZEB1 in the samples with good prognosis, it was observed that in approximately 33% of the samples, ZEB1 was absent (G and H), with ITIH2 being moderate in one of these samples (G) and absent in the other (H); and in approximately 66% of the samples, ZEB1 showed strong staining (B, D, I, J), whereas ITIH2 also stained strongly in one (approximately 16.66%) of these samples (B) and was absent in 50% of them (D, I, J). In contrast, in malignant tumors with poor prognosis, n = 4, ITIH2 staining ranged from moderate in 50% of the samples (A, E) to absent in 50% (C, F), whereas ZEB1 showed strong intensity in all of these samples. Double staining with ENO1 and ZEB1 (Figure 7) revealed distinct patterns. In tumors with good prognosis, ENO1 was present in all samples, ranging from moderate in approximately 16.6% of samples (H) to strong in approximately 83% of samples (B, D, I, J, G), accompanied by strong ZEB1 staining in approximately 33% of samples (B, D) and absence of ZEB1 in approximately 66.6% of these samples (I, J, G, H). However, in malignant tumors with reserved prognosis, ENO1 showed moderate staining in approximately 50% of samples (E, F) and strong in the other 50% (A, C), while ZEB1 showed consistently strong intensity.

### 5.5. Predictive Potential

**Figure 9:**
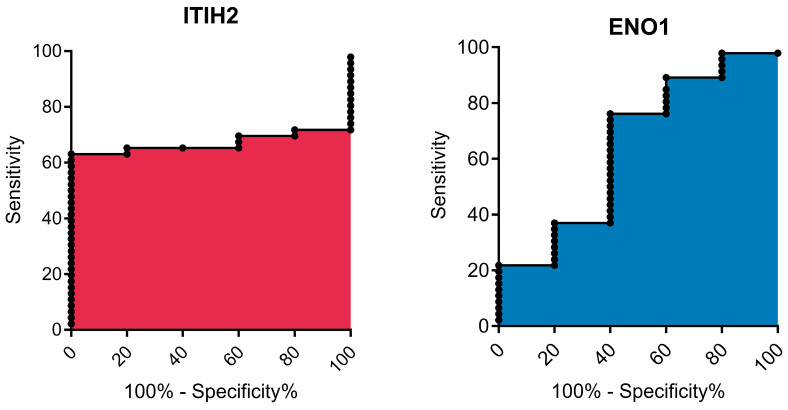
Roc curves of ITIH2 (Inter-Alpha-Trypsin Inhibitor Heavy Chain 2) and ENO1 (Enolase 1).

The prognostic potential of ITIH2 and ENO1 (Figure 9) for mammary tumors in bitches was assessed using ROC curve analysis, which revealed limited predictive power. For ITIH2, the area under the curve (AUC) was 0.67 ± 0.07 (95% confidence interval (CI) of 0.54–0.80; *p* = 0.217). The optimal cutoff value was (<44.50), providing a sensitivity of 65.22% (CI: 49.75–78.65%) and a specificity of 80% (CI: 28.36–99.49%). ENO1 exhibited an AUC of 0.64 ± 0.14 (95% CI: 0.37–0.92; *p* = 0.296), with a cutoff value of <127.9 yielding a sensitivity of 76.09% (CI: 61.23–87.41%) and a specificity of 60% (CI: 14.66–94.73%).

## 6. Discussion

The malignant group exhibited a higher mean age compared to the benign group, consistent with findings reported in the literature [27]. Conversely, tumor size did not differ significantly between the two groups, a result that contrasts with previous studies [28]. Anatomopathological characteristics such as necrosis, anisoacaryosis, nuclear pleomorphism, and increased mitotic activity were more pronounced in the malignant group, in alignment with established literature [29].

As a secreted protein, ITIH2 is expected to be present in both normal and tumor tissue, as well as in various locations, including plasma within vessels and in the extracellular medium. This widespread distribution complicates the establishment of a direct relationship between ITIH2 and breast tumors, as it is observed in all samples across groups. To overcome this challenge, a specific staining criterion was adopted: staining was considered positive only when present within tumor cells. In our findings, no intense ITIH2 staining was detected within cells in any sample. Moderate to weak cytoplasm staining was observed in some cases, with rare nuclear staining. This weak intracellular presence suggests that there is another function of ITIH2 besides its primary one, in the extracellular matrix and plasma. However, its occasional intracellular detection warrants further investigation into what its potential additional roles are, especially in cancer biology. Despite these results highlighting a limited cellular presence of ITIH2, its role in inflammation and extracellular matrix stabilization suggests potential relevance to breast cancer prognosis [30]. ITIH2 appears to have a dual, context-dependent function in tumor biology. On the one hand, its antiproteolytic function and regulation of the extracellular matrix (ECM) may suppress tumor progression. On the other hand, interactions with molecules such as hyaluronic acid and modulation of the inflammatory microenvironment may exert ambivalent effects, depending on the tumor’s stage and context. A comprehensive understanding of ITIH2’s role in tumors requires considering the specific cancer type, disease stage, and the surrounding microenvironment.

The expression of ITIH2 in breast tumors can be explained by its binding with hyaluronic acid, a molecule overexpressed in malignant tumors. Interestingly, hyaluronic acid serum levels do not increase in patients with localized breast tumors but rise during advanced disease stages [31]. In contrast, studies have shown reduced hyaluronic acid levels in infiltrating breast tumors [32,33]. Hyaluronic acid cross-linking with ITIH2 via tumor necrosis factor-induced protein 6 (TSG6) reduces its degradation and alters extracellular matrix (ECM) interactions, which may offer cancer-protective effects [34]. Although our discussions on ITIH2 are primarily based on studies of human breast cancer, this is due to the lack of specific data for dogs. This gap underscores the need for future investigations exploring the role of this protein in canine mammary tumors, contributing to a better understanding of its relevance in this context.

Regarding ENO1, the fact that there were no differences between the groups draws attention to the specificity of the antibody used. A higher frequency of simultaneous cytoplasmic and nuclear staining was observed in the same cell in all groups, especially in the control group, which exclusively presented this type of staining; in the benign group, there was mostly simultaneous staining of the cytoplasm and nucleus, but there was also staining only in the cytoplasm in some samples; in the malignant group, there was a lot of simultaneous staining of the cytoplasm and nucleus, but in some samples it stained only the cytoplasm, and in others it stained the plasma membrane. Cytoplasmic staining is in line with its role as a glycolytic enzyme, while nuclear localization—reported predominantly in benign tumors—is linked to its isoform MBP-1, which binds to C-myc, inactivating it, thus acting as a tumor suppressor [35]. However, cytoplasmic staining does not always predict ENO1, and it may be MBP1, its isoform.

In malignant cells, there was simultaneous staining of the cytoplasm and nucleus, as well as staining of the cell membrane, which did not occur in the benign group or in the control group. This fact corroborates studies that report the expression of ENO1 on the cell surface exceptionally in malignant tumors, where it binds to plasminogen to promote ECM degradation and metastasis [36]. Our findings using the anti-ENO1 antibody revealed expression of both ENO1 (cytoplasm/membrane) and MBP1 (nucleus and cytoplasm). A study showed that both proteins can be expressed in normal and tumoral mammary glands [37]. Our results show that in all groups there was a majority staining of the nucleus and cytoplasm, with additional expression of the membrane, exclusive to the malignant tumor group.

This shows that concomitant staining of ENO1 and MBP1 may have occurred in all groups, but we were unable to differentiate them because we used an antibody that recognizes ENO1 and its isoform MBP1. We interpret this as stating that it is necessary to use antibodies specific for ENO1 and another for MBP1; only then could we be certain of the quantifications related to each one, as well as knowing which of the two isoforms would be in the cytoplasm, since both can be expressed in the cytoplasm. Corroborating this, an article [37] stated that the studies conducted so far in tumor and normal tissue samples depend on the use of commercially and non-commercially available antibodies recognizing both ENO1 and MBP-1, which has made it difficult to clearly assess the relative expression of the two proteins by immunohistochemistry. We hypothesize that if there is greater expression of ENO1 to the detriment of MBP1, this could evidence energetic favoritism for the tumor; on the other hand, if there is majority expression of MBP1, it could be inferred that the tumor cells are in hypoxia, since MBP1 does not have an enzymatic function in glycolysis like ENO1. As a conclusion regarding the expression of ENO1, we can state that we are certain of its membrane expression exclusively in malignant tumors.

The low AUC values indicate that ITIH2 and ENO1 have limited predictive capability. The wide confidence intervals and lack of statistical significance further highlight their limited utility as individual prognostic markers. These findings underscore the need for larger studies to confirm these results and suggest that combining biomarkers or exploring alternative analytical approaches may yield better predictive performance. Further research into the biological roles of ITIH2 and ENO1 is also warranted.

In canine mammary tumors, foreign tissues such as bone and cartilage are commonly observed due to epithelial–mesenchymal transition. Knowing that ZEB1 regulates the transcription of ITIH2, the additional test we performed with ZEB1 aimed to investigate its presence for two reasons: the first was to find out whether there is a relationship between ZEB1 and ITIH2 in canine mammary tumors, and the second was to confirm whether ZEB1 regulates the transcription of ITIH2 [38]. Our results regarding the immunohistochemical test with ZEB1 and visual analysis showed that this protein is indeed present in both benign and malignant mammary tumors of bitches. However, it is important to clarify that statistically the number of samples, both total and distributed in the subgroups “good prognosis and reserved prognosis”, was insufficient. However, our objective was only to collaborate with future studies, verifying the absence or presence of ZEB1 and whether this coincided with the intensity of staining of ITIH2 and ENO1, as well as the absence/presence of these two proteins, all this to suggest future studies of colocalization/co-expression of these proteins in mammary tumors of bitches. It was observed that in tumors with good prognosis, ITIH2 presented absent to moderate staining, while ZEB1 presented intense staining in most samples, although absent in a small part; in tumors with reserved prognosis, ITIH2 did not differ in its staining pattern, but ZEB1 stained intensely and strongly in all samples.

In double staining with ENO1 and ZEB1, we observed that in tumors with good prognosis, while ENO1 was marked strongly in most samples, ZEB1 was absent. In tumors with reserved prognosis, it was observed that ZEB1 was present with strong intensity in all samples. Moreover, ENO1 was also present in all samples, but was ranging from moderate to strong intensity. In summary, in mammary tumors of dogs, ZEB1 was found in tumors with good prognosis and in those with poor prognosis but showed a tendency to be present and to mark more intensely in tumors with poor prognosis; in addition, its expression showed no association with ITIH2, but it did with ENO1.

CD44 is a transmembrane glycoprotein that acts as an endocytic HA receptor in human breast tumor cells [39]; therefore, future investigations should explore the interactions between ITIH2, hyaluronic acid, and CD44 in canine and human breast tumors, given the evidence of potential functional relationships. Furthermore, ZEB1 could also be included in these investigations.

Regarding ENO1, future studies with larger sample sizes are essential to elucidate its interaction with ZEB1 and its subcellular localization in tumor cells, as these factors provide significant information on tumor progression and prognosis.

## 7. Conclusions

This study investigated the immunohistochemical expression of ITIH2 and ENO1 in mammary tissues from healthy dogs and those with benign and malignant tumors. Although no statistically significant differences in histoscores were observed between groups, we identified two findings: ENO1 staining of the plasma membrane was exclusive to malignant tumors, and its nuclear presence was observed in both benign and malignant tumors, contradicting previous reports of its restriction to benign cases. Furthermore, we confirmed the presence of the MBP-1 (nuclear) isoform in normal mammary tissue, highlighting its relevance beyond tumor contexts.

Although this study faced limitations, including sample size, our findings open avenues for exploring the mechanisms underlying cancer in the canine species. Notably, the involvement of ITIH2 and ENO1 in extracellular vesicles (EVs) and their prevalence in the bloodstream highlight their potential as biomarkers and therapeutic targets. These results highlight the importance of further research into the role of these proteins in cancer progression, with implications for veterinary and comparative oncology.

## Figures and Tables

**Table 1:** Anatomopathological profile of the malignant, benign, and control groups.

	Malignant	Benign	Control	*p*
**Necrosis n (%)**				
Absent	14 (37)	19 (50)	05 (13)	0.027 *
Moderate	01 (100)	0 (0)	0 (0)	
Central	2 (100)	0 (0)	0 (0)	
Frequent	08 (100)	0 (0)	0 (0)	
Abundant	01 (100)	0 (0)	0 (0)	
Accentuated	01 (100)	0 (0)	0 (0)	
**Anisokaryosis n (%)**				
Absent	17 (47)	14 (39)	05 (14)	0.005 *
Discret	0 (0)	05 (100)	0 (0)	
Moderate	07 (100)	0 (0)	0 (0)	
Frequent	03 (100)	0 (0)	0 (0)	
**Nuclear pleomorphism n (%)**				
Absent	02 (17)	05 (42)	05 (42)	<0.001 *
Discret	05 (36)	09 (64)	0 (0)	
Moderate	13 (72)	05 (28)	0 (0)	
accentuated	07 (100)	0 (0)	0 (0)	
**Tumor diameter (cm), mean ± SE (n)**	1.34 ± 0.87 (11)	0.76 ± 0.40 (17)	–	0.063 †
**Lymphatic invasion, n (%)**				
Absent	22 (54)	19 (46)	–	0.067 *
Present	05 (100)	0 (0)	–	
**Metastasis, n (%)**				
Absent	23 (55)	19 (45)	–	0.131 *
Present	19 (100)	0 (0)	–	

SEM = standard error of the mean. * *p*-values for Fisher’s exact test. † *p*-value for a *t*-test with Welch’s correction.

## Data Availability

To ensure transparency and accessibility of data, we provide a table with the Histoscore data and anatomopathological profile of all patients, and another table with the metadata.

## Supplementary Materials

The following supporting information can be downloaded at: https://www.mdpi.com/article/10.3390/vetsci12020110/s1, To ensure transparency and accessibility of data, we provide a table with the Histoscore data and anatomopathological profiles of all patients and another table with the metadata.
